# Differentiating between Near- and Non-Cognate Codons in *Saccharomyces cerevisiae*

**DOI:** 10.1371/journal.pone.0000517

**Published:** 2007-06-13

**Authors:** Ewan P. Plant, Phuc Nguyen, Jonathan R. Russ, Yvette R. Pittman, Thai Nguyen, Jack T. Quesinberry, Terri Goss Kinzy, Jonathan D. Dinman

**Affiliations:** 1 Department of Cell Biology and Molecular Genetics, University of Maryland, College Park, Maryland, United States of America; 2 Department of Molecular Genetics, Microbiology and Immunology, University of Medicine and Dentistry of New Jersey (UMDNJ) Robert Wood Johnson Medical School, Piscataway, New Jersey, United States of America; 3 The Science and Technology Center at Eleanor Roosevelt High School, Greenbelt, Maryland, United States of America; 4 Huntington High School, Huntington, Maryland, United States of America; University of Florida, United States of America

## Abstract

**Background:**

Decoding of mRNAs is performed by aminoacyl tRNAs (aa-tRNAs). This process is highly accurate, however, at low frequencies (10^−3^ – 10^−4^) the wrong aa-tRNA can be selected, leading to incorporation of aberrant amino acids. Although our understanding of what constitutes the correct or cognate aa-tRNA:mRNA interaction is well defined, a functional distinction between near-cognate or single mismatched, and unpaired or non-cognate interactions is lacking.

**Methodology/Principal Findings:**

Misreading of several synonymous codon substitutions at the catalytic site of firefly luciferase was assayed in *Saccharomyces cerevisiae*. Analysis of the results in the context of current kinetic and biophysical models of aa-tRNA selection suggests that the defining feature of near-cognate aa-tRNAs is their potential to form mini-helical structures with A-site codons, enabling stimulation of GTPase activity of eukaryotic Elongation Factor 1A (eEF1A). Paromomycin specifically stimulated misreading of near-cognate but not of non-cognate aa-tRNAs, providing a functional probe to distinguish between these two classes. Deletion of the accessory elongation factor eEF1Bγ promoted increased misreading of near-cognate, but hyperaccurate reading of non-cognate codons, suggesting that this factor also has a role in tRNA discrimination. A mutant of eEF1Bα, the nucleotide exchange factor for eEF1A, promoted a general increase in fidelity, suggesting that the decreased rates of elongation may provide more time for discrimination between aa-tRNAs. A mutant form of ribosomal protein L5 promoted hyperaccurate decoding of both types of codons, even though it is topologically distant from the decoding center.

**Conclusions/Signficance:**

It is important to distinguish between near-cognate and non-cognate mRNA:tRNA interactions, because such a definition may be important for informing therapeutic strategies for suppressing these two different categories of mutations underlying many human diseases. This study suggests that the defining feature of near-cognate aa-tRNAs is their potential to form mini-helical structures with A-site codons in the ribosomal decoding center. An aminoglycoside and a ribosomal factor can be used to distinguish between near-cognate and non-cognate interactions.

## Introduction

Accurate transmission of biological information is a central requirement at all levels of life. In cells, one aspect of this process is the faithful translation of the genetic code from DNA into protein. The intermediaries in the last stage of this process include mRNA, tRNAs, ribosomes and many *trans*-acting factors. The protein coding information of an mRNA is formatted as codons. The anticodon loops of aminoacyl tRNAs (aa-tRNAs) form base-pairing interactions with the codons. This enables ribosomes to add amino acids sequentially to the nascent protein. aa-tRNAs that can participate in standard Watson-Crick interactions with the first two bases in a codon and can form either canonical or non-Watson-Crick pairs at the third or “wobble” position are designated cognate-tRNAs [1], [2]. In contrast, tRNAs that do not meet these requirements are commonly referred to as near- and non-cognate tRNAs. Utilization of near- and non-cognate tRNAs is called misreading or a missense error. Misreading occurs with low frequencies of 10^−3^ and 10^−4^ per codon ([3] and references within).

**Figure 1:**
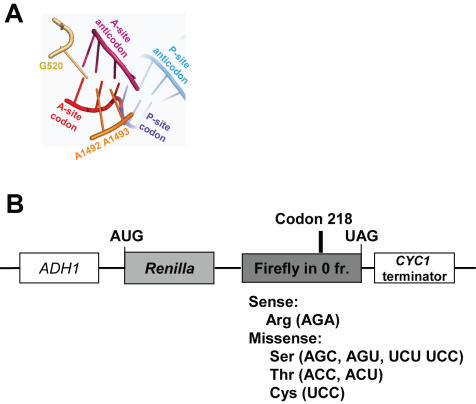
The decoding center and dual-luciferase reporters for determining rates of translational misreading in yeast. Panel A. The codon:anticodon mini-helix in the decoding center is stabilized by base-pairing at all three positions of the mini-helix favoring A-minor interactions with flipped out bases G520, A1492 and A1493. PyMol (Delano Scientific, LLC) was used to generate this figure based on the coordinates 1IBM in the RSCN Protein Data Bank [40]. Panel B. In all missense reporters, transcription is initiated from the yeast *ADH1* promoter, and terminated at a sequence from the *CYC1* 3′ UTR. The luciferase genes from *Renilla* and firefly are cloned in frame to produce a fusion of the two proteins. The sense reporter has the AGA codon encoding arginine at amino acid residue 218 in the catalytic site of firefly luciferase. Missense reporters contain the indicated mutations at this position, which encode the indicated amino acids. Efficiencies of missense suppression were calculated by dividing the ratio of firefly/Renilla luciferase generated from cells harboring the missense test vectors by the ratio of firefly to Renilla luciferase generated from cells harboring the sense control plasmid.

The 64 codons encode 20 different amino acids and three termination signals. In cases where one amino acid is represented by multiple codons, some tRNAs can decode more than one codon. This redundancy is facilitated by tRNA modifications and by wobble base-pairing between the anticodon and the codon (reviewed in [4]). Our understanding of how the ribosome achieves such a high degree of specificity has been facilitated by both kinetic and structural analyses in bacteria (reviewed in [5]–[7]). *In vitro* kinetic analyses using ribosomes, tRNAs, and the bacterial aa-tRNA binding factor EF-Tu have broken down the process of aa-tRNA selection into a series of discrete steps (reviewed in [6] ). These studies have identified two stages (k_−2_ and k_7_) in this process that favor rejection of aa-tRNAs whose anticodon loops cannot base-pair with codons. A mutational analysis demonstrated that two independent mechanisms corresponding to these two steps are required for utilization of cognate aa-tRNAs [8]. Structural, biophysical, and computational analyses also show a mechanism for positive selection of cognate aa-tRNAs[7]–[10]} that emphasizes the geometry of base pairing at the ribosomal decoding center [11]. Formation of an appropriately configured mini-helix in the decoding center generates an RNA minor-groove, enabling interaction with three critical bases of the small subunit rRNA. Formation of this mini-helix stimulates A1492 and A1493 of the small subunit rRNA to flip out into the minor groove forming a complex arrangement of hydrogen bonds with the tRNA/mRNA backbones in concert with G530 (Figure 1A). This in turn stimulates a conformational change in the aa-tRNA that transduces the information from the decoding center to activate the GTPase activity of EF-Tu (reviewed in [12]). The energy barrier for flipping out of A1492 and A1493 is sufficiently small for correct binding of aa-tRNA to shift the equilibrium in favor of the subsequent steps [7]. Aminoglycoside antibiotics such as paromomycin stimulate misreading by binding to the decoding center, displacing A1492 and A1493. This forces these bases to mimic the “flipped out” conformations that they normally assume in response to the mini-helix formation by a cognate codon:anticodon pair (reviewed in [5]). Together, these kinetic and biophysical mechanisms ensure the accurate utilization of cognate aa-tRNAs.

An unsettled issue remains the precise distinction between “near-cognate” and “non-cognate” tRNAs, especially in eukaryotes. This is important since the rational design or utilization of therapeutics may exploit the functional differences that exist between these two classes of tRNAs. One recent study has suggested that the relative abundances of bacterial aa-tRNAs plays a significant role in translational accuracy [3]. By this model, highly abundant aa-tRNAs are more likely than low abundance aa-tRNAs to misread codons that are decoded by other low abundance aa-tRNAs. The current study using the yeast *Saccharomyces cerevisiae* supports this competition model. However it also suggests that this is not sufficient to explain the functional differences between near- and non-cognate aa-tRNAs. Using a series of seven substitutions of a codon in the catalytic site of firefly luciferase, we show that a second critical distinction lies in the ability to form hydrogen bonding interactions at all three positions between the aa-tRNA anticodon loop and the codon in the decoding center. This is likely the result of changes in formation of the codon:anticodon mini-helix. Thus, transient formation of the mini-helix allows the rRNA and tRNA conformational changes required for activation of the GTPase activity of eEF1A, the eukaryotic homolog of EF-Tu. This is supported by the demonstration of paromomycin stimulated misreading by aa-tRNAs that are capable of forming a transient interaction. This identifies paromomycin as a functional probe to distinguish between near- and non-cognate aa-tRNAs. The hypothetical roles in this process played by eEF1A and its associated factors, eEF1Bα and eEF1Bγ, were also investigated. The results suggest that the GTPase activity of eEF1A is preferentially stimulated by near-cognate codon:anticodon interactions, and point to discrete functional regions of the protein. Studies of eEF1Bα, the catalytic subunit of the guanine nucleotide exchange factor (GEF) required for recycling of GTP-bound eEF1A, are consistent with a kinetic model in which limiting concentrations of eEF1A:aa-tRNA:GTP ternary complex should decrease rates of protein synthesis, resulting in increased selection against both near- and non-cognate aa-tRNAs. Interestingly, deletion of the proposed regulatory subunit of the GEF complex, eEF1Bγ, tended to promote increased misreading of near-cognate codons and decreased misreading of non-cognate codons. This suggests eEF1Bγ may have a regulatory function. In a final series of experiments, the potential role of the fungus-specific elongation factor eEF3 in translational fidelity was indirectly assayed through analysis of a series of mutants in the ribosomal protein L5 (rpL5). rpL5 forms part of the ribosome binding site for this factor [13]. Like the eEF1Bα mutant, the ability of an rpL5 mutant to promote enhanced fidelity at both non- and near-cognate codons suggests that slowing rates of elongation by disrupting the synergy between eEF1A and eEF3 results in increased selection against both near- and non-cognate aa-tRNAs.

## Results

### Baseline and paromomycin-stimulated rates of missense suppression suggests a functional difference between near- and non-cognate tRNAs

Introduction of a missense mutation into the active site of an enzyme followed by quantitative measurement of the restoration of enzymatic activity can provide a basis to monitor translational error rates. The arginine at position 218 of firefly luciferase is located in the active site and is required for enzymatic activity. Mutation of the corresponding AGA arginine codon to either the AGC or UCU serine codon was previously used to monitor missense error rates of ribosome bound chaperone mutants in *S. cerevisiae* [14]. The current study employed a bicistronic luciferase reporter system to monitor suppression of a series of missense mutations. This could functionally distinguish translational fidelity effects due to the inherent translatability of different codons and subsequently correlate these with *trans*-acting influences. In this assay, the gene encoding firefly luciferase is fused in frame with a downstream *Renilla* luciferase gene. Test plasmids harbored missense codons at position 218 of the firefly luciferase gene (Figure 1B, “Missense”). The control was identical except that it contained the wild-type AGA codon at this position (Figure 1B, “Sense”). Even though the two luciferase proteins are fused, the activity from each can be measured independently as they utilize different substrates. Rates of misreading were calculated by dividing the ratio of firefly luciferase activity to *Renilla* luciferase activity generated from the missense vector in strains harboring the indicated mutant allele by the ratio generated with the sense plasmid. The results were statistically tested as previously described [15].

Previous studies have shown that missense errors occur with frequencies on the order of 10^−4^ in both *E. coli* and in *S. cerevisiae* [3], [16]–[18]. Consistent with the literature, the seven missense mutations assayed at position 218 demonstrated ratios of firefly to *Renilla* luciferase activities that were reduced by approximately 4 orders of magnitude in all cases (Table 1). Although rates of misreading are low and the differences between test and control samples are small, the sensitivity of the assay and rigor of the statistical methods enable meaningful analysis of the data. Inspection of these data revealed that rates of misreading varied over an approximately 4.5-fold range. The inability of any one amino acid (i.e. Ser, Cys, or Thr) to disproportionately influence apparent missense incorporation suggests that the assay monitored incorporation errors rather than altered luciferase activity arising from incorporation of any specific amino acid. The data reveal that the common element among the three most stimulatory codons is the potential to form a stable G•C base pair at the second position. The observation of different error rates among the four serine codons (which are decoded by different tRNA families) suggests that relative tRNA abundances are also important.

**Table 1:** Baseline levels and effects of paromomycin on suppression of missense mutations at codon 218 of firefly luciferase.

	Mis-Incorporation Freq. (×10^−4^)			
218 codon alleles	No Drug	+ Paromomycin	Fold Change	P-value
AGC Ser	6.53±0.42	8.36±0.53	1.30	0.01
AGU Ser	2.88±0.17	3.81±0.21	1.30	2.55E-3
UGU Cys	3.56±0.28	4.89±0.28	1.40	2.99E-3
UCU Ser	2.23±0.09	2.33±0.09	1.00	0.46
UCC Ser	2.27±0.13	2.43±0.14	1.10	0.42
ACC Thr	1.92±0.29	1.55±0.06	0.81	0.25
ACU Thr	1.37±0.05	1.32±0.08	1.00	0.60

Wild-type codon is AGA Arginine.

**Figure 2:**
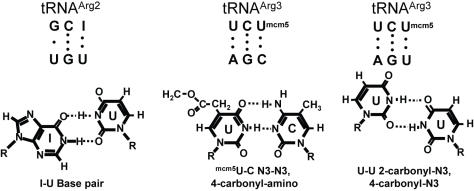
Proposed basis for near-cognate codon-anticodon interactions. Top. Base pairing between tRNA^Arg2^ anticodon and UGU codon (left), and between tRNA^Arg3^ anticodon and the AGC and AGU codons. Bottom. I•U, U•C, and U•U base pairing.

Formation of the mini-helix in the decoding center stimulates flipping out of the small subunit bases A1492 and A1493 that in combination with G530 stabilizes this structure (Figure 1A). Paromomycin binding to the decoding center of the small ribosomal subunit displaces A1492 and A1493, thus enhancing the frequency of missense errors (reviewed in [6]). It has been hypothesized that not only does the formation of the mini-helix displace A1492 and A1493, but once displaced they sterically position the interribose bonds to maintain A-form helices [19]. In the current study, translational misreading errors were significantly stimulated by paromomycin at codons that are capable of forming complete mini-helices with arginyl-tRNAs: UGU, AGU and AGC (Figure 2). In contrast, paromomycin did not stimulate misreading with anticodons unable to form the mini-helix, e.g. UCU, UCC, ACU and ACC. These findings suggest a functional definition for near- versus non-cognate codon:anticodon interactions. This potential to form base pairing interactions at all three positions, which is possibly nucleated by a strong Watson-Crick base pair at the second position would allow transient formation of the mRNA:tRNA mini-helix. We propose this as the defining feature of a near-cognate interaction. In contrast, non-cognate interactions are defined by their lack of potential to form the mini-helix.

### eEF1A mutants generally affect utilization of near-cognate aa-tRNAs

**Figure 3:**
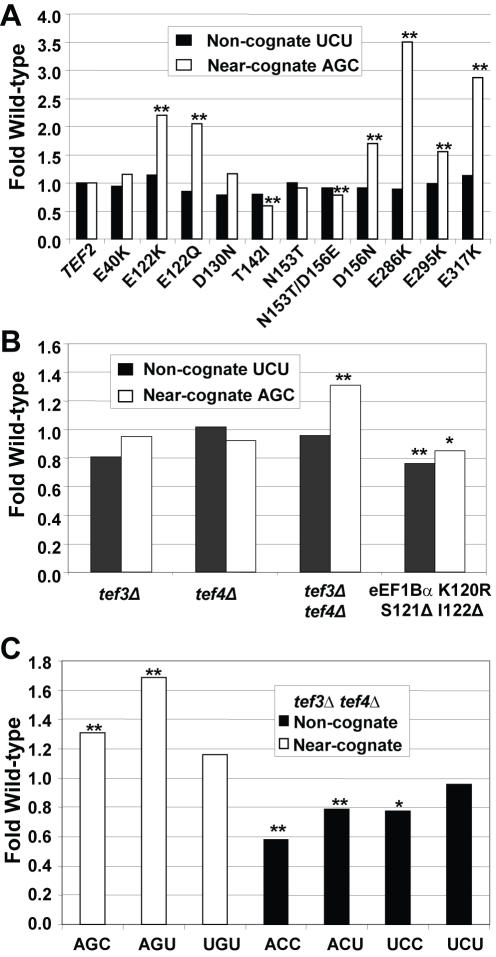
Effects of the eEF1 complex mutants on mis-reading of near- and non-cognate codons. Misreading of the non-cognate UCU and near-cognate AGC codons by mutant forms of eEF1A (Panel A), or by isogenic strains with *tef3Δ, tef4Δ*, or *tef3Δ tef4Δ* double null mutants or *tef5Δ* strains expressing the K120R S121Δ I122Δ allele compared to isogenic wild-type strain (Panel B). Panel C. Misreading of all seven missense codons by cells lacking both forms of eEF1Bγ (*tef3Δ tef4Δ*). Effects of the indicated mutants are depicted as fold of isogenic wild-type cells. ** indicates *p* values of <0.01; * indicates *p* values of <0.05.

In eukaryotes, a ternary complex composed of eEF1A, aa-tRNA, and GTP delivers the aa-tRNA to the A-site of the ribosome. When an aa-tRNA containing the correct anticodon is sampled by the ribosome, a signal is transmitted through the body of the tRNA. This stimulates GTP hydrolysis by eEF1A and subsequent accommodation of the tRNA into the ribosomal A-site (reviewed in [5], [6]). eEF1A is encoded by the essential *TEF1* and *TEF2* genes in *S. cerevisiae*. A set of *TEF2* mutants expressed in a *tef1Δ tef2Δ* genetic background [20] were assayed with respect to their effects on misreading using the AGC and UGU serine codons to monitor misreading of near- and non-cognate codons, respectively. The results show allele-specific responses specifically to near-cognate codons. Strains bearing one of six alleles (E122K, E122Q, D156N, E286K, E295K, and E317K) promoted enhanced misreading of the near-cognate AGC Ser codon, but not of non-cognate UCU Ser (Figure 3A, Table 2, p<0.01). In contrast, a strain expressing the T142I and to the lesser extent N153T/D156E mutant were better able to distinguish between the cognate AGA and near-cognate AGC codons. This enhanced fidelity did not extend to the non-cognate UCU codon (Figure 3A, Table 2, p<0.01). In summary, it appears that near-cognate codons and not non-cognate are able to influence eEF1A activity in an allele-specific manner.

**Table 2:** Effects of selected alleles of genes on mis-reading of non-cognate and near-cognate mutations at codon 218 of firefly luciferase.

	Non-cognate (UCU)			Near-cognate (AGC)		
Strain	mis-incorp (×10^−4^)	Fold WT	P-value	mis-incorp.(×10^−4^)	Fold-WT	P-Value
**eEF1A**						
WT (*TEF2*)	2.04±0.13	1.00	1.00	7.01±0.49	1.00	1.00
E40K (*TEF2-3*)	1.91±0.18	0.94	0.56	8.11±0.72	1.16	0.22
E122K (*TEF2-4*)	2.33±0.17	1.14	0.17	15.4±0.55	2.20	9.76E-10
E122Q (*TEF2-10*)	1.73±0.16	0.85	0.14	14.4±0.79	2.05	2.43E-6
D130N (*TEF2-13*)	1.61±0.12	0.79	0.02	8.16±0.21	1.16	0.05
T142I (*TEF2-7*)	1.62±0.14	0.79	0.03	4.20±0.48	0.60	6.64E-4
N153T (*tef2-19*)	2.05±0.04	1.00	0.95	6.39±0.14	0.91	0.06
N153T/D156E (tef*2-18*)	1.87±0.05	0.91	0.03	5.53±0.20	0.79	4.92E-3
D156N (*tef2-17*)	1.85±0.04	0.91	0.02	11.94±0.39	1.69	3.10E-7
E286K (*TEF2-1*)	1.81±0.08	0.89	0.04	24.64±0.11	3.50	3.58E-8
E295K (*TEF2-9*)	2.00±0.20	0.98	0.87	10.9±0.95	1.55	3.09E-3
E317K (*TEF2-2*)	2.31±0.04	1.13	0.06	20.1±0.91	2.87	1.70E-7
**eEF1Bα**						
WT	3.15±0.12	1.00	1.00	3.50±0.14	1	1.00
K120R S121Δ I22Δ	2.38±0.22	0.76	8.89E-3	2.98±0.10	0.85	0.01
**eEF1Bγ**						
WT	1.22±0.14	1.00	1.00	1.24±0.09	1	1.00
*tef3Δ*	1.00±0.04	0.81	0.15	1.18±0.05	0.95	0.49
*tef4Δ*	1.25±0.14	1.02	0.41	1.14±0.06	0.92	0.41
*tef3Δ tef4Δ*	1.17±0.05	0.96	0.62	1.62±0.03	1.31	1.09E-5

### A mutation in eEF1Bα promotes general hyperfidelity

After hydrolysis of GTP, the eEF1A:GDP complex is released from the ribosome. The eEF1Bα subunit (encoded by *TEF5*) is the essential nucleotide exchange factor responsible for catalytic activity in eEF1A recycling [21]. Analysis of the role of the eEF1Bα protein capitalized on the availability of the K120R S121Δ I122Δ mutant form of the protein that altered or deleted residues involved in critical interactions with the nucleotide binding pocket of eEF1A [22]. This mutant strain was previously shown to enhance translational fidelity by promoting lower levels of readthrough at all three stop codons [23]. In the current study, the K120R S121Δ I122Δ eEF1Bα mutant showed modest but consistent enhanced fidelity at both the UCU and AGC non- and near-cognate codons respectively (Figure 3B, Table 2, p<0.01). As discussed below, we hypothesize that this may be due to limiting concentrations of eEF1A:GTP.

### Codon-specific misreading in the absence of eEF1Bγ

In yeast, the eEF1 complex contains a third non-essential subunit, eEF1Bγ. Yeast cells express two isoforms of eEF1Bγ, encoded by the *TEF3* and *TEF4* genes. The N-terminus of Tef3p exhibits structural similarity to gluthathione-S-transferases and is thought to participate in the regulation of elongation during stress [24], [25]. Deletion of either eEF1Bγ isoform alone had no significant effects on misreading at either the AGC near-cognate or UGU non-cognate codons (Figure 3B, Table 2). However deletion of both eEF1Bγ-encoding genes caused a statistically significant increase in mis-incorporation at near- but not at non-cognate codons (Figure 3B, Table 3). These results suggest that these two proteins have redundant activities and that this factor functions in ensuring translational fidelity. To investigate this phenomenon further, misreading in the absence of eEF1Bγ (*tef3Δtef4Δ*) was assayed at the remaining 5 missense codons. Although misreading of the near-cognate AGU Ser codon was also enhanced, there was no effect on recognition of the ‘near-cognate’ UGU Cys codon (Table 3, Figure 3C). In contrast, although recognition of the UCU ‘non-cognate’ codon was not affected, recognition of the other non-cognate codons (ACC, ACU, and UCC) was significantly enhanced by the absence of eEF1Bγ.

**Table 3:** Survey of isogenic wild-type and mutant pairs of strains with seven different codons at codon 218 of firefly luciferase.

codon	mis-incorp (×10^−4^)		Fold WT	P-value
**eEF1Bγ**				
	**WT**	***tef3Δtef4Δ***		
AGC	9.78±0.31	12.8±0.23	1.31	1.1E-05
AGU	2.85±0.21	4.81±0.11	1.69	4.1E-06
UGU	2.81±0.16	3.27±0.18	1.16	0.08
ACC	1.39±0.06	0.80±0.06	0.58	2.2E-05
ACU	1.13±0.05	0.89±0.05	0.79	3.0E-03
UCC	2.07±0.08	1.60±0.14	0.78	0.02
UCU	3.03±0.25	2.90±0.13	0.96	0.62
***RPL5***				
	**WT**	**K27E**		
AGC	10.6±0.51	9.39±0.80	0.88	0.21
AGU	3.26±0.08	2.81±0.05	0.86	0.05
UGU	2.95±0.14	2.50±0.18	0.85	0.02
ACC	1.16±0.07	0.95±0.04	0.82	0.02
ACU	0.99±0.05	0.76±0.02	0.77	1.0E-03
UCC	1.59±0.11	1.75±0.06	1.11	0.22
UCU	2.47±0.15	1.33±0.09	0.54	1.5E-07

### The K27E mutant of ribosomal protein L5 promotes a general enhancement of fidelity

**Figure 4:**
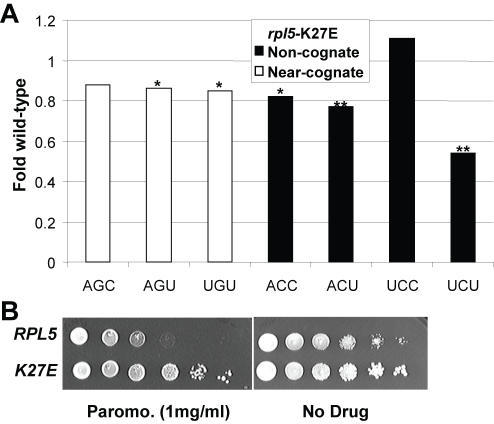
Characterization of alleles of *RPL5.* Panel A. Effects of the K27E rpl5 mutanton misreading of seven missense codons. Effects on the indicated missense reporters are depicted as fold of isogenic wild-type cells. ** indicates p values of <0.01. Panel B. Paromomycin dilution spot assays. Ten-fold dilutions (10^6^→10^1^ CFU) of logarithmically growing cells were arrayed onto H-leu medium containing paromomycin (1 mg/ml) or no drug control plates. Cells were grown at 25°C for 3 days.

The three-site model of the ribosome posits that preventing simultaneous occupancy of the ribosomal A- and E-sites by aa-tRNA and deacylated-tRNAs respectively helps to coordinate the elongation cycle (reviewed in [26]). In fungi, the unique essential elongation factor 3 (eEF3) facilitates eEF1A-dependent A-site binding of aa-tRNA and has ATP-dependent activity required for the release of deacylated tRNA from the E site [27]. Although ribosomal protein L5 (rpL5) is far from the decoding center, it has been shown to interact with eEF3 [13]. Previous studies have characterized five temperature-sensitive alleles of the yeast *RPL5* gene encoding rpL5 [28], [29]. A preliminary assay of five *rpl5* mutant strains using the near- (AGC) and non- (UCU) cognate reporters indicated that the K27E mutant of L5 tended to be generally hyperaccurate (data not shown). Analysis of all 7 missense codons revealed that the K27E mutant generally promoted greater levels of translational accuracy (Figure 4A, Table 3). Dilution spot assays revealed that rpL5-K27E is resistant to paromomycin (Figure 4B), consistent with the notion that this mutant is antagonistic to the action of the drug. As discussed below, these findings suggest an indirect role for rpL5 in translational decoding. This may be through its association with eEF3 and account for some differences in A-site fidelity between bacteria and fungi

## Discussion

A significant number of human diseases are caused by missense mutations. Understanding the functional differences between mutations and drugs that result in near- versus non-cognate codon usage may inform strategies for therapeutic interventions. In the current study, codon misreading was examined in the yeast eukaryotic model system using a dual luciferase reporter in which the AGA Arg codon in the firefly luciferase catalytic site was changed to AGC, AGU, UCU, UCC (serine); UGU (cysteine); and ACC or ACU (threonine). Each of these substitutions has a polar sidechain like arginine, but lacks the positive charge. The consistent 4-order of magnitude decrease in firefly luciferase activity with these substitutions agrees with previously characterized missense error rates. The finding no one amino acid alters firefly luciferase activity outside a range supports that the assay monitors misincorporation of arginine. The observation that the frequency of Arg misincorporation varied over an approximately 4.5-fold range independent of the identity of the encoded amino acid suggests that misincorporation frequency is determined by other factors.

In each species, some codons are used more frequently than others. The codon bias is especially clear in highly expressed genes. Studies in *E. coli* have led to the suggestion that codon bias minimizes the deleterious effects of aberrant decoding [30], [31]. Examination of codon bias in highly expressed yeast genes (see [32]) reveals that both the AGU and AGC Ser codons occur at a low frequency of approximately 5% of serine in the yeast genome. All of the other codons employed in this study occur with significantly higher frequencies. UGU is the most frequently used Cys codon at 84% in highly transcribed genes. Its substitution promoted even higher rates of miscoding than AGU Ser at 5% codon frequency. Thus, it is clear that codon frequency is not the sole determinant of translational fidelity. A recent study in *E. coli* suggested that competition between different tRNAs is the underlying factor influencing misreading error rates [3]. In yeast, gene copy number for individual tRNA species correlates with initial estimates of relative tRNA content in normally growing cells, allowing the number of functional genes encoding cognate tRNAs for each codon to be used as a proxy measure of tRNA abundance [33], [34]. Examination of yeast tRNA gene copy numbers [35] reveals that AGA Arg is decoded by the highly abundant tRNA^Arg3^ (11 copies). In support of the tRNA competition model, the codons that intrinsically promoted higher levels of misreading were decoded by lower abundance tRNAs, while those at the lower end of the range are decoded by more abundant tRNAs. For example, the AGU and AGC codons are decoded by the relatively low abundance tRNA^Ser3^ (4 copies), and all Cys codons are decoded by the 4 copy tRNA^Cys^. In contrast, the UCU and UCC codons are decoded by tRNA^Ser2^ (11 copies), and the ACC and ACU codons are decoded by the high copy tRNA^Thr1a^ (11 copies). However, tRNA competition alone cannot fully explain the observed differences. The pattern becomes more apparent when the ability of near-cognate codons to base pair with anticodons of different arginyl-tRNAs is considered (Figure 2). The first two bases of the AGC and AGU Ser codons could be recognized by the ^mcm5^UCU anticodon of the highly abundant tRNA^Arg3^. Base pairing at the wobble positions of these two codons and ^mcm5^U is also theoretically possible through N3-N3, 4-carbonyl-amino, and 2-carbonyl-N3, 4-carbonyl-N3 hydrogen bonding respectively (see Figure 2). Note that although there are three possible U•U base pairs and two possible C•U couples, the geometries of the N3-N3, 4-carbonyl-amino, and of the 2-carbonyl-N3, 4-carbonyl-N3 hydrogen pairing schemes provide the most energetically favorable topologies within the constraints of the tRNA:mRNA mini-helix. Further, it has been suggested that cmo^5^ modification of U_34_ stabilizes the shape of the anticodon loop (reviewed in [4]). Although the C1′-C1′ distance between pyrimidine-pyrimidine nucleotide pairs is short, and is thus destabilizing relative to that for pyrimidine-purine base pairs, biophysical analyses suggest that bridging water molecules could produce stable and planar U_34_•U3 and U_34_•C3 base pairs (reviewed in [4]). Of note, although biophysical analyses of RNA duplexes indicate that U•U base-pairing is more stable than U•C pairs at pH 7.0, the observation that the AGC codon promoted>2-fold more misreading than the AGU codon suggests that this particular U•C base pair is more energetically permissible within the topological constraints imposed by the codon:anticodon mini-helix structure. Examination of the UGU Cys codon also reveals that it can potentially base pair at all three positions with the ICG anticodon of tRNA^Arg2^, which is encoded by 6 genes (Figure 2). Although G_36_•U1 pairing does not normally occur in cognate codon:anticodon interactions, this has been posited to occur at the P-site in Ty*1* promoted programmed +1 ribosomal frameshifting [36]. The lower abundance of this tRNA in combination with the less stable G_36_•U1 base pair at the first position of this pair may account for the lower frequency of misreading of this codon as compared to AGC Ser. tRNA modifications that facilitate wobble position interactions are present in both bacterial and eukaryotic systems, supporting the hypothesis that the formation of a mini-helix may be central for the ribosome to distinguish between near- and non-cognate interactions across the kingdoms. Kramer and Farabaugh also noted that misreading was enhanced in the presence of paromomycin for codons where there was potential for U•U base-pairing [3]. However, the frequency of misreading varies between eukaryotes and bacteria indicating that other trans-acting factors or ribosomal components are involved. We extended our analysis to examine the effects of some of these factors.

### Influence of eEF1A and associated elongation factors in translational fidelity

Because eEF1A delivers aa-tRNA to the ribosome and cognate codon:anticodon interactions stimulate its GTPase activity, mutants of this factor could alter translational fidelity in two ways. First, altered affinity for the ribosome could affect the initial binding step. Second, changes in intrinsic GTPase activity could affect aa-tRNA stimulation threshold. Because initial binding is independent of codon:anticodon interactions, mutants affecting the first step would be expected to alter fidelity in response to both near- and non-cognate codons. Since such an outcome was not observed for any of the eEF1A mutants, it is unlikely that any of the mutants affected initial binding rates. In contrast, since codon:anticodon interactions between near-cognate aa-tRNAs might be more likely to induce tRNA structural changes than those between non-cogante aa-tRNAs, eEF1A mutants with increased intrinsic GTPase activity or with decreased activation thresholds would more likely be stimulated by near-cognate as opposed to non-cognate aa-tRNAs. Similarly, those having decreased intrinsic GTPase activity or increased activation thresholds would be more discriminatory when presented with near-cognate aa-tRNAs.

**Figure 5:**
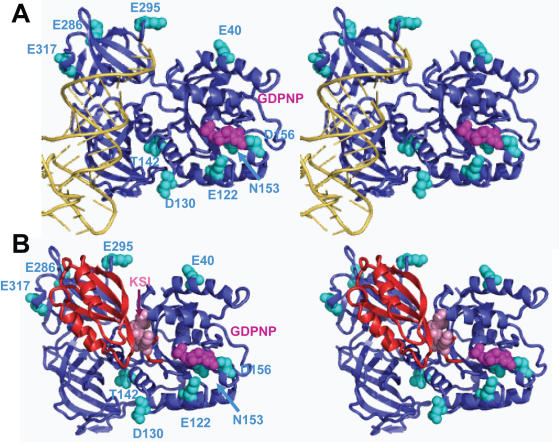
Modeling of mutations in eEF1A and eEF1Bα that influence influence misincorporation of missense aa-tRNAs. The nucleotide exchange factor eEF1Bα and the fitted aa-tRNA present clashes indicating that they do not interact with eEF1A simultaneously. PyMOL (Delano Scientific, LLC) was used with the coordinates 1G7C of yeast eEF1A:eEF1Bα (amino acids 114–206) in complex with GDPNP [50]. The ribbon structure of eEF1A is shown in blue, eEF1A mutated bases are shown in cyan, and GDPNP is indicated in magenta. Panel A. tRNA (yellow) was fitted into the structure based on coordinates obtained from the crystal structure of the EF-Tu:Phe-tRNA^Phe^:GCPNP complex (1TTT in the RSCN Protein Data Bank, [51]. Panel B. Ribbon structure of eEF1Bα from 1G7C is shown in red, and the KSI residues in the mutant form used in this study are indicated in salmon.

Examination of the mutants within the context of the structure of the eEF1A•aa-tRNA•GTP modeled as the ternary complex provides some clues with regard to which mechanism may be altered in these mutants (Figure 5A). The charge reversal mutants in domain 2 proposed to be involved in binding the tRNA acceptor stem (E286K, E295K and E317K) all promoted enhanced misreading of the near-cognate AGC codon. It is tempting to speculate that these mutants may promote increased aa-tRNA dissociation rates by mimicking the structural change induced by correct tRNA:mRNA interactions and subsequently stimulating the GTPase activity of eEF1A. In contrast, the T142I mutant in domain 1 that interacts with the phosphate backbone of residue 61 at the base of the D-loop was more discriminatory, suggesting that this interaction is important for stimulation of GTP hydrolysis. Mutants in the vicinity of the GTP binding pocket had allele-specific effects on incorporation of the near-cognate tRNA. Charge reversal or neutralization of E122K or D156N strongly stimulated misreading. Perhaps the presence of additional positive charge in this region enhances GTP binding and/or GTPase activity. In contrast, loss of a positive charge in the N153T mutant slightly inhibited misreading. Curiously, this effect was enhanced in the N153T/D156N double mutation. Last, mutations of E40 and D130 which are not closely linked to either tRNA or GTP binding did not affect missense suppression even though they were initially isolated as +1 insertion suppressor mutants. It is also striking that genetic screens have never identified fidelity mutations in domain 3, which is also proposed to interact with aa-tRNA. This suggests that these interactions are either non-essential or irrelevant to presentation at the A-site.

The K120R S121Δ I122Δ (KSI) mutant of eEF1Bα (*tef5–7*) promoted increased accuracy in decoding both the ACG near- and UCU non-cognate codons. As noted above, mutants affecting fidelity in response to both near- and non-cognate codons are likely to be due to altered affinity at the initial binding step. Previous genetic analyses showed that this mutant also promoted increased fidelity at nonsense codons, slower rates of growth and protein synthesis, and hypersensitivity to translational inhibitors [23]. Figure 5B shows the X-ray crystal structure of eEF1A in complex with the catalytic terminus of eEF1Bα. eEF1Bα binds to domain I and II of eEF1A, where KSI amino acids are located close to the nucleotide-binding pocket in domain 1 of eEF1A. The binding site of eEF1Bα to domain II overlaps the proposed aa-tRNA binding site. This is significantly different from the bacterial homolog, and may be another way in which eukaryotes and bacteria differ in maintaining fidelity and promoting aa-tRNA delivery. We propose that these residues may facilitate exchange of GDP for GTP by eEF1A. Slowing this process would serve to limit the availability of functional ternary complexes, thus affecting binding of both near- and non-cognate codons. The effects of loss of eEF1Bγ shows a general trend of enhanced accuracy of non-cognate codons like an eEF1Bα mutant. However, the increased misreading of a near-cognate codon is more like an eEF1A mutant. Thus, although the precise function of eEF1Bγ has yet to be determined, it is reasonable to hypothesize that it may modulate eEF1Bα to affect fidelity.

### Ribosomal protein L5: Coordination of tRNA exit from the E-site with aa-tRNA entrance at the A-site

The observation that the rpL5-K27E mutant generally promoted increased accuracy was initially surprising since this protein is located on the back of the central protuberance of the large subunit, far away from the ternary complex binding site, the ribosomal A-site, and the decoding center. In addition, a previous study implicated rpL5 in binding of peptidyl-tRNA, but not of aa-tRNAs [29]. Nonetheless, the rpL5-K27E mutant generally promoted increased fidelity in response to almost all of the near- and non-cognate codons tested. An intriguing explanation for the allele-specific effects observed here may come from the observation that rpL5-L5 interacts with the fungal-specific elongation factor eEF3 [13]. eEF3 is an ATPase that interacts with eEF1A and catalyzes release of deacylated-tRNA from the ribosomal E-site [27], [37]. It has been proposed that eEF3 and eEF1A work synergistically to remove deacylated tRNAs from the E-site and promote delivery of cognate aa-tRNA to the A-site [27]. It is tempting to speculate that altered eEF3 binding to the K27E form of rpL5 upsets this synergy. Similar to the model proposed for the eEF1Bα KSI mutant, the rpL5-K27E mutant might promote enhanced accuracy at the codon recognition step.

It should also be noted that a previous study also used a dual luciferase reporter system to examine missense suppression in yeast [17]. The analyses applied in the current study cannot be directly applied to the data generated by Salas-Marco and Bedwell [17] since that work employed eight different codons at two positions of firefly luciferase. Since most were non- synonymous, the effects of different amino acid substitutions and their specific locations on the activity of the enzyme cannot be controlled for. Since different strain backgrounds are used in the studies and even in different sets of mutants analyzed in this work, variations in wild-type values observed requires the use of specific statistical methodology for accurate comparisons between datasets [15]. Further, relative tRNA abundance issues complicated the two instances where synonymous codons were used. Despite these differences, independent analyses, reporter constructs and strain backgrounds showed similar levels of misreading. Furthermore, specific effects on misreading were observed for mutants of ribosomal components and key factors involved in elongation and termination. These data support the universal application of this approach to studies of translational fidelity.

### Kinetics: the difference between near- and non-cognate interactions may occur at the GTPase activation step

As described above, studies in the bacterial system shows accuracy during translation elongation is likely a two step process involving two distinct biophysical mechanisms: initial selection and proofreading (reviewed in [5], [38]). The first step, initial binding, is mostly determined by the interaction between the ribosome and EF-Tu, and forward and reverse rates (k_1_ and k_−1_) are not affected by aa-tRNA identity [6]. However, during the next step of codon recognition, the stabilizing effects due to interactions of G530, A1492 and A1493 with the mini-helix results in dissociation rates (k_−2_) of near-cognate aa-tRNAs being approximately 400-fold than those of cognate aa-tRNAs [39]. Computational modeling suggests the existence of two major energy minima at the decoding center corresponding to the flipped-in and flipped-out conformations of A1492, and A1493, and that fast flipping between the two states provides a kinetic means to discriminate at the level of codon:anticodon interactions [10]. Formation of the stable mini-helix results in the physical transduction of information to the ternary complex, thus activating the endogenous GTPase of EF-Tu (k_3_). This step acts as a kinetic trap to select for aa-tRNAs capable of forming the mini-helix. We propose that it is here that the difference between near- and non-cognate tRNA:mRNA interactions also occurs in eukaryotes. The potential of near-cognate interactions to form mini-helices, albeit at lower frequencies, provides the opportunity for stimulation of GTPase activation. The data also suggest that the presence of a canonical Watson-Crick base pair between N_35_•N′2 may aid in nucleating mini-helix formation, consistent with molecular dynamics modeling showing that stability testing by the kink in the mRNA between the P- and A-site codons destabilizes position 2 mismatches more severely than mismatches at the first position [9]. In contrast, non-cognate interactions cannot possibly form mini-helices, and thus are incapable of forming the kinetic trap and activating GTP hydrolysis. In a kinetic analysis comparing different codon:anticodon mismatches, one tRNA capable of participating in a non-cognate interaction was employed and stimulated GTPase activation approximately 6.7 fold less than the near-cognate codons [39]. This is consistent with the ∼4.5 fold increased rates of misreading promoted by near-cognate codons in the current study.

### Using paromomycin to functionally distinguish between ‘near-’ and ‘non-cognate’ codon:anticodon interactions

Binding of cognate aa-tRNA stimulates rearrangement of G530, A1492, and A1493 to establish A-minor interactions between themselves and the minor groove of the codon-anticodon helix [11], [40]. Binding of paromomycin to the decoding center stimulates similar displacement of A1492 and A1493, positioning them to stabilize codon:anticodon interactions in a promiscuous manner [41], perhaps trapping them in the flipped-out state [10]. In light of the data presented here, we suggest that mini-helix formation is a precondition for paromomycin-stimulated misreading. Furthermore, paromomycin-enhanced and the potential for mini-helix formation are coordinately maximized in the decoding center. Thus, paromomycin has the potential to be used as a tool to functionally distinguish between ‘near-’ and ‘non-cognate’ codons. However, this requires expansion of the system to utilize other codons for which the encoded amino acids do not result in a partially active luciferase protein. Kramer and Farabaugh observed changes in misreading with two different aminoglycosides, paromomycin and streptomycin [3]. Although more information about aminoglycoside-ribosome interactions has recently become available (reviewed in [42]) more experimental work needs to be performed to determine if any aminoglycosides other than paromomycin may be better sensors of cognate status.

## Materials and Methods

### *E. coli* and yeast strains and genetic methods

*E. coli* strain DH5α was used to amplify plasmids. High efficiency transformations were performed as previously described [43]. The *Saccharomyces cerevisiae* strains used in this study are listed in Table 4. The *RPL5* strains were generously provided by Dr. John Woolford. Isogenic *TEF2, TEF3*, and *TEF4* yeast strains were previously described [25], [44], [45]. Strains were cultured on YPAD or synthetic complete medium (H-) [46] and were freshly plated and incubated for two to five days at 30°C prior to transformation. Yeast were transformed with the alkali cation method [47], plated on appropriate selective media, and incubated at 30°C for four days. To assay for paromomycin sensitivity, 10-fold dilutions of logarithmically growing cells were spotted onto H-leu containing drug at a concentration of 1 mg/ml, or onto no-drug control plates, and grown at 25°C for 3 days.

**Table 4:** Yeast Strains used in this study

Strain	Genotype	Source
JD932D	*MATa ade 2-1 trp1-1 ura3-1 leu2-3,112 his3-11,15 can1-100* [L-AHN M_1_]	[52]
M213	*MATα leu2-3,112 his4-713 ura3-52 trp1Δ lys2-20 met2-1 tef2Δ tef1::LEU2+ pTEF2*	[20]
TKY111	*MATα leu2-3,112 his4-713 ura3-52 trp1Δ lys2-20 met2-1 tef2Δ tef1::LEU2+pTEF2-2* E317K	[53]
TKY112	*MATα leu2-3,112 his4-713 ura3-52 trp1Δ lys2-20 met2-1 tef2Δ tef1::LEU2+pTEF2-3* E40K	[53]
TKY113	*MATα leu2-3,112 his4-713 ura3-52 trp1Δ lys2-20 met2-1 tef2Δ tef1::LEU2+pTEF2-4* E122K	[53]
TKY114	*MATα leu2-3,112 his4-713 ura3-52 trp1Δ lys2-20 met2-1 tef2Δ tef1::LEU2+pTEF2-7* T142I	[53]
TKY115	*MATα leu2-3,112 his4-713 ura3-52 trp1Δ lys2-20 met2-1 tef2Δ tef1::LEU2+pTEF2-9* E296K	[53]
TKY116	*MATα leu2-3,112 his4-713 ura3-52 trp1Δ lys2-20 met2-1 tef2Δ tef1::LEU2+pTEF2-10* E122Q	[53]
TKY117	*MATα leu2-3,112 his4-713 ura3-52 trp1Δ lys2-20 met2-1 tef2Δ tef1::LEU2+pTEF2-13* D130N	[53]
TKY278	*MATα leu2-3,112 his4-713 ura3-52 trp1Δ lys2-20 met2-1 tef2Δ tef1::LEU2+pTEF2-17* D156N	[23]
TKY280	*MATα leu2-3,112 his4-713 ura3-52 trp1Δ lys2-20 met2-1 tef2Δ tef1::LEU2+pTEF2-19* N153T	[23]
TKY282	*MATα leu2-3,112 his4-713 ura3-52 trp1Δ lys2-20 met2-1 tef2Δ tef1::LEU2+pTEF2-18* N153T D156E	[23]
TKY539	*MATα leu2-3,112 his4-713 ura3-52 trp1Δ lys2-20 met2-1 tef2Δ tef1::LEU2+pTEF2-1* E286K	This work
TKY677	*MATa ura3-52 trp1Δ101 lys2-801 his3Δ200 leu2Δ1*	[25]
TKY678	*MATa ura3-52 trp1Δ101 lys2-801 his3Δ200 leu2Δ1 tef3::LEU2*	[25]
TKY679	*MATa ura3-52 trp1Δ101 lys2-80 his3Δ200 leu2Δ1 tef4::TRP1*	[25]
TKY680	*MATa ura3-52 trp1Δ101 lys2-801 his3Δ200 leu2Δ1 tef3::LEU2 tef4::TRP1*	[25]
TKY235	*MATα ura3-52 trp1Δ101 lys2-801 leu2Δ1 met2-1 his4-713 tef5::TRP1 pTEF5 URA3*	[23]
TKY243	*MATα ura3-52 trp1Δ101 lys2-801 leu2Δ1 met2-1 his4-713 tef5::TRP1 pTEF5-7 K120R S121Δ I22Δ*	[23]
JWY3742	*MATα ura3-52 trp1-Δ101 leu2 his3-Δ200 ade1 rpl5-Δ1::TRP1+pRS315-RPL1-HA* (*RPL5*)	[28]
JWY3750	*MATα ura3-52 trp1-Δ101 leu2 his3-Δ200 ade1 rpl5-Δ1::TRP1+pRS315-RPL1-HA-1* (*rpl5- K27E*)	[28]
JWY3751	*MATα ura3-52 trp1-Δ101 leu2 his3-Δ200 ade1 rpl5-Δ1::TRP1+pRS315-RPL1-HA-2* (*rpl5-T28A*)	[28]
JWY3749	*MATα ura3-52 trp1-Δ101 leu2 his3-Δ200 ade1 rpl5-Δ1::TRP1+pRS315-RPL1-HA-3* (*rpl5-V53G*)	[28]
JWY3752	*MATα ura3-52 trp1-Δ101 leu2 his3-Δ200 ade1 rpl5-Δ1::TRP1+pRS315-RPL1-HA-4* (*rpl5-G91R*)	[28]
JWY3761	*MATα ura3-52 trp1-Δ101 leu2 his3-Δ200 ade1 rpl5-Δ1::TRP1+pRS315-RPL1-HA-5* (*rpl5-K289E*)	[28]

### Plasmid Constructs

Plasmids used in this study contained a dual luciferase cassette on a yeast vector backbone with the *URA3* selectable marker. The parental pYDL-Control plasmid containing the wild-type *Renilla* and firefly luciferase genes has been described previously [48]. Missense mutations were introduced into the arginine codon (AGA) at position 218 in the catalytic site of firefly luciferase [14] using variations on the following primers:

5′-ATGCGAGAANNNGACGCAGGCAGTTCTATG-3′ and 5′-GCCTGCGTCN′N′N′TTCTCGCATGCCAGAGATC-3′ (Integrated DNA Technology, Coralville, IA) where N denotes bases at codon 218 that were changed on the sense strand, and N′ are the corresponding bases mutagenized on the antisense strand. Oligonucleotide site directed mutagenesis reactions were performed using the StrataGene Quikchange II kit (La Jolla, CA) according to the manufacturer's instructions. The seven mutants thus created are listed in Table 1. A second set of plasmids containing the yeast *TRP1* reporter were constructed by transferring the dual luciferase cassettes from the resulting plasmids as Spe*I*–Xho*I* fragments into p414 ADH [49].

### Dual Luceriferase Assays

Transformed yeast cells were grown overnight in selective medium at 30°C to OD_595_ of 0.8 to 1.0. Cells were pelleted by centrifugation, washed twice with 0.5 ml of cold lysis buffer (phosphate buffered saline containing 1 mM phenylmethylsulfonylfluoride), resuspended in cold lysis buffer and broken by agitation with glass beads (0.5 mm BioSpec). Lysates were clarified by centrifugation, and supernatants transferred to pre-chilled tubes. Luminescence reactions were initiated by addition of 50 µl of Promega DLR system to 5 µl of clarified cell lysates and measured using a Turner Design TD20/20 luminometer. At least three readings were taken for each assay and all assays were repeated (n = 3 – 12) until the data were normally distributed to enable statistical analyses both within and between experiments [15]. An unpaired two-sample *t*-test was used to test the hypothesis that two datasets came from the same population, a rejected hypothesis indicating that the datasets were significantly different. The *P*-values from this test is the estimation of the probability of an incorrect conclusion [15].
